# Directional Persistence of Cell Migration in Schizophrenia Patient-Derived Olfactory Cells

**DOI:** 10.3390/ijms22179177

**Published:** 2021-08-25

**Authors:** Jing Yang Tee, Alan Mackay-Sim

**Affiliations:** 1 Griffith Institute for Drug Discovery, Griffith University, Nathan, QLD 4111, Australia; teejy@szbl.ac.cn; 2 BayRay Innovation Center, Shenzhen Bay Laboratory, Guangming District, Shenzhen 518106, China

**Keywords:** schizophrenia, disease modeling, patient-derived cell model, cell migration, live cell imaging, extracellular matrix, ECM

## Abstract

Cell migration is critical for brain development and linked to several neurodevelopmental disorders, including schizophrenia. We have shown previously that cell migration is dysregulated in olfactory neural stem cells from people with schizophrenia. Although they moved faster than control cells on plastic substrates, patient cells were insensitive to regulation by extracellular matrix proteins, which increase the speeds of control cells. As well as speed, cell migration is also described by directional persistence, the straightness of movement. The aim of this study was to determine whether directional persistence is dysregulated in schizophrenia patient cells and whether it is modified on extracellular matrix proteins. Directional persistence in patient-derived and control-derived olfactory cells was quantified from automated live-cell imaging of migrating cells. On plastic substrates, patient cells were more persistent than control cells, with straighter trajectories and smaller turn angles. On most extracellular matrix proteins, persistence increased in patient and control cells in a concentration-dependent manner, but patient cells remained more persistent. Patient cells therefore have a subtle but complex phenotype in migration speed and persistence on most extracellular matrix protein substrates compared to control cells. If present in the developing brain, this could lead to altered brain development in schizophrenia.

## 1. Introduction

The correct development of the brain is critically dependent on cell migration. During early embryonic development, newborn cells move in highly coordinated patterns [1] and require directional persistence to reach their intended destinations [2,3]. Directional persistence is the tendency for migrating cells to keep moving in the same direction without turning. The efficiency of cells exploring their extracellular environment depends on directional persistence and on the speed of cell migration [4]. Neuronal migration in developing and adult brains, such as cortical lamination, are regulated in part by extracellular matrix (ECM) proteins [5]. Dysregulated responses to guidance cues affect the tangential migration of cortical interneurons [6] and neuronal precursors migrating to the olfactory bulb as well as the radial migration of granule cells in the cerebellum [7].

Multiple lines of evidence suggest that cell migration may be altered in schizophrenia, leading to changes in brain development and subsequent illness. Disrupted cell migration is implicated in the thinner cortex in post-mortem brains in schizophrenia [8], which may arise from reduced interneuron migration during development in schizophrenia [9,10]. There is dysregulation of expression of genes involved in cell migration in schizophrenia patient-derived post-mortem brain tissues [11] and induced pluripotent (iPS) cell-derived neural progenitor cells [12,13,14]. In vitro functional studies indicate that cell migration is affected in patient-derived iPS cell-derived neural progenitor cells [12] and in cerebral organoids generated from patient-derived, iPS cell-derived neurons [15].

We have developed a model to study cell migration in schizophrenia based on primary cultures of human olfactory neurosphere-derived (ONS) cells, a neural stem cell from the olfactory mucosa, the organ of smell [16]. We have accumulated considerable evidence that schizophrenia patient-derived ONS cells have multiple deficits in functions associated with cell adhesion and migration, compared to ONS cells from healthy controls. Patient cells were less adhesive, were smaller than control cells, and had less filamentous actin and fewer stable microtubules [17,18]. Key cytoskeletal components actin, microtubules, and focal adhesions were dysregulated in the transcriptomes of schizophrenia patient ONS cells, many of which are intracellular regulators of directional persistence and turning during cell migration, including 38 genes from the focal adhesion kinase (FAK) signaling pathway [19]. Patient cells migrated faster on a fibronectin substrate [17] and had smaller and fewer focal adhesions with faster disassembly than control cells [17]. Patient cell motility was correlated with cell adhesion and levels of phosphorylated FAK (pFAK) [17]. Interestingly, patient cells did not regulate their migration speed in response to extracellular matrix proteins (ECM proteins), unlike control cells [18]. Patient cells migrated at the same speeds on all ECM proteins; they were faster than control cells on tissue culture plastic but control cell migration speeds increased with ECM protein concentration until control cell speeds equaled patient cell speeds, or surpassed them [18]. These deficits were not due to a lack of ability to detect ECM proteins: patient and control cells had similar integrins, and patient cells, like control cells, increased the number and size of focal adhesions upon stimulation by ECM proteins [18]. Taken together, these observations suggest that patient cells have deficits or biases in the dynamic regulation of cell migration, as if detection of the ECM is decoupled from the molecular mechanisms controlling cell migration speed. 

The dynamics of cell migration are best described by a suite of quantitative measures, such as distance traveled, migration speed, area explored, turning behavior, and directional persistence [4,20]. Directional persistence is an exponential function of migration speed [4] that is modified by intracellular and extracellular factors [4,20]. Directional persistence is regulated through dynamic regulation of actin filament branching and elongation that steers the extension and persistence of lamellipodia [21]. We hypothesized that, like migration speed, directional persistence is altered in schizophrenia patient-derived cells. The aim of the present study was to quantify multiple measures of directional persistence in the migration of schizophrenia patient ONS cells and control ONS cells on different extracellular substrates. For this study, we reanalyzed our cell migration datasets that demonstrated speed differences in patient and control cells [18] to quantify directional persistence [20], cell idling, and cell turning [22]. The variables calculated were: “directionality” (how straight were the trajectories of cell movement), “diffusion” (how much of the microenvironments were explored), “directional persistence” (the tendency to maintain movement in the same direction), “idling time” (the period spent stationary), and the “turn angle” (the angle of change of direction) [20,22]. 

In this study, we demonstrated that patient cells are different from controls in all the measures of directional persistence and turning behaviors. These deficits, if present during brain development, could plausibly alter the characteristics of neuronal migration trajectories in schizophrenia. The intrinsic bias of persistence in patient cells was affected by different ECM substrates, in contrast to cell migration speed, indicating further complexity to the dysregulation of cell migration in schizophrenia. These phenotypes provide tools for understanding the molecular mechanisms of this dysregulation and potentially for the discovery of drugs to ameliorate it.

## 2. Results

### 2.1. Patient Cells Moved in Straighter Trajectories than Control Cells at Baseline

**Figure 1:**
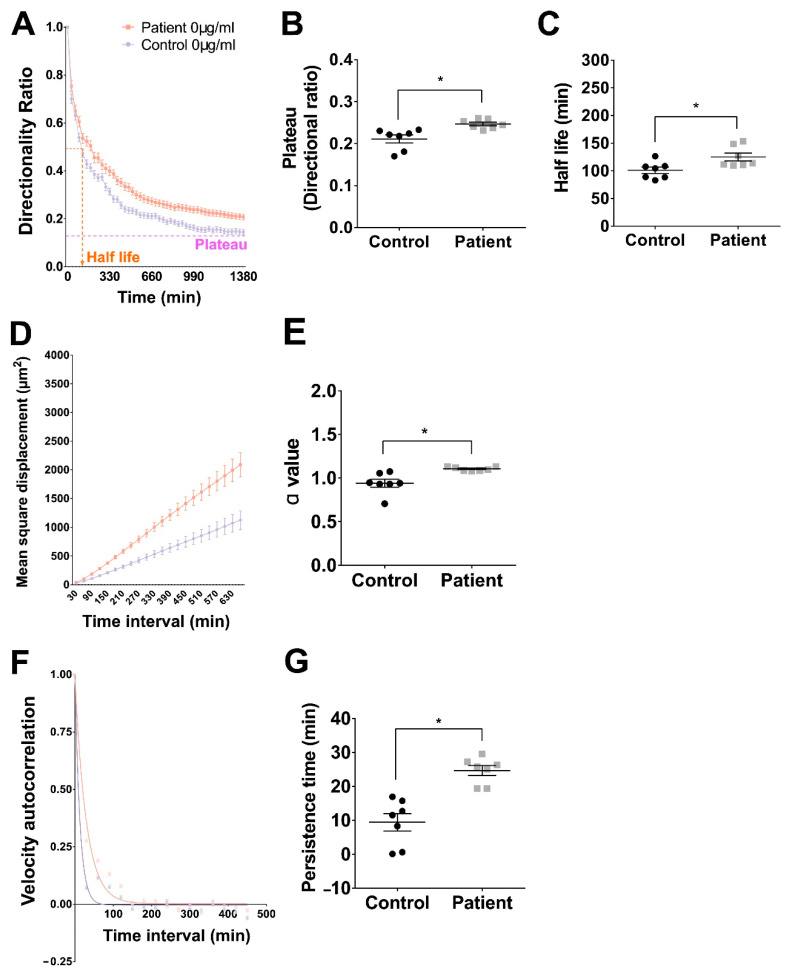
Patient cells were more persistent than control cells on uncoated tissue culture plastic. (**A**): Directionality ratio calculated for patient cells (red) and control cells (blue). Mean ± SEM at each time point. Indications of Plateau and Half-life are shown. (**B**): Plateau values for patient and control cells. Symbols represent means of all cells for individuals in each group. Bars represent mean ± SEM for each group. (**C**): Half-life values for patient and control cells. Symbols represent means of all cells for individuals in each group. Bars represent mean ± SEM for each group. (**D**): Mean Square Displacement calculated for patient cells (red) and control cells (blue). Mean ± SEM at each time point. (**E**): MSD α-values for patient and control cells. Symbols represent means of all cells for individuals in each group. Bars represent mean ± SEM for each group. (**F**): Velocity autocorrelation calculated for patient cells (red) and control cells (blue). Mean ± SEM at each time point. (**G**): Persistence times (inverse of decay rate of velocity autocorrelation) for patient and control cells. Symbols represent means of all cells for individuals in each group. Bars represent mean ± SEM for each group. * = *p* < 0.05, *t*-test.

A directionality ratio of 1 indicates a cell is moving in a straight line, while a ratio of 0 indicates random movement at a particular measured point. The directionality ratio decays exponentially from 1 at time = 0 min, to a plateau value (Figure 1A). Data were fitted to a one-phase decay function to quantify two parameters: Plateau and Half-life (DRPlateau, DRHalf-life, Figure 1A). At baseline conditions on uncoated tissue culture plastic (TCP), patient cells had a plateau value significantly higher than control cells (Patient: 0.211 ± 0.0095, *n* = 7; Control: 0.247 ± 0.0042, *n* = 7) (Figure 1B; *p* = 0.023, *t* = 2.62, df = 12). At baseline conditions on TCP, patient cells had a significantly longer half-life value than control cells (Patient: 125.0 ± 7.13 min, *n* = 7; Control: 101.0 ± 5.76 min; *n* = 7) (Figure 1C; *p* = 0.023, t = 2.62, df = 12).

### 2.2. Patient Cell Migration Covered Greater Surface Areas than Control Cells at Baseline

The surface area that a single cell explores in a given period is described by mean square displacement (MSD). MSD increases with time (Figure 1D). At baseline on TCP, patient cells (red line) explored a larger surface area than control cells (blue line). MSD–time curves were transformed into log–log curves whose slope, α, characterizes the different patterns of cell movement: α = 1 represents random movement and α = 2 represents fully directional movement. At baseline on TCP, patient cells had a significantly larger α value than control cells (MSD, Patient: α = 1.11 ± 0.0084, *n* = 7; Control: α = 0.94 ± 0.045; *n* = 7) (Figure 1E; *p* = 0.0038, *t* = 3.57, df = 12).

### 2.3. Patient Cells Spent More Time in Persistent Movement than Control Cells at Baseline

Velocity autocorrelation estimates how cell velocity in a time interval correlates with its velocity in the previous time interval. The velocity autocorrelation decays exponentially from a starting value of 1 at time = 0. (Figure 1F). Velocity autocorrelation curves were fitted using a one-phase decay function (Figure 1F). The inverse of the rate of decay is the persistence time, a measure of directional persistence. At baseline on TCP, patient cell velocity autocorrelation curves (red line; Figure 1F) decayed slower than control cell curves (blue line; Figure 1F). Baseline persistence times were significantly longer in patient cells compared to control cells (Patient: 24.71 ± 1.48 min, *n* = 7; Control: 9.45 ± 2.57 min, *n* = 7) (Figure 1G; *p* = 0.0002, *t* = 5.14, df = 12).

### 2.4. Patient Cells Spent More Time in Active Movement and Less Time Idling than Control Cells at Baseline

**Figure 2:**
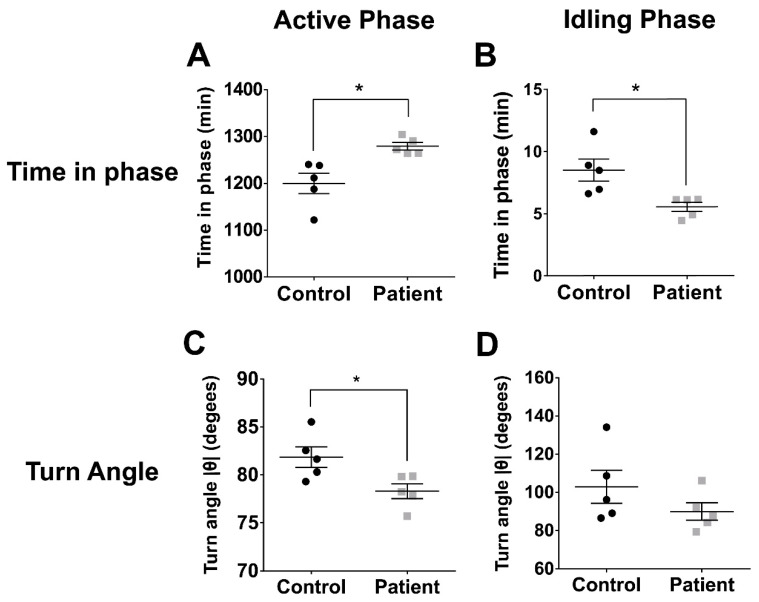
Patient cells were more active and made smaller turns than control cells on uncoated tissue culture plastic. (**A**): Time spent in Active Phase: Symbols represent means of all cells for individuals in each group. Bars represent mean ± SEM for each group. (**B**): Time spent in Idling Phase: Symbols represent means of all cells for individuals in each group. Bars represent mean ± SEM for each group. (**C**): Turn Angle following Active Phase: Symbols represent means of all cells for individuals in each group. Bars represent mean ± SEM for each group. (**D**): Turn Angle following Idling Phase: Symbols represent means of all cells for individuals in each group. Bars represent mean ± SEM for each group. * = *p* < 0.05, *t*-test.

Cell movement was divided into the active and idling phases to estimate the times spent in each phase during the period of observation. At baseline levels on TCP, patient cells spent significantly more time in the active phase of migration than control cells (Patient: 1279 ± 8.1 min, *n* = 5; 1200 ± 21.8 min, *n* = 5) (Figure 2A; *p* = 0.0092, *t* = 3.42, df = 8). In a reciprocal manner, patient cells spent significantly less time in the idling phase than control cells (Patient: 5.55 ± 0.36 min, *n* = 5; Control: 8.51 ± 0.89 min, *n* = 5) (Figure 2B; *p* = 0.015, *t* = 3.09, df = 8).

### 2.5. Patient Cells Made Smaller Turns during the Active Phase of Cell Migration than Control Cells at Baseline

The turning tendencies of each cell were quantified as the turn angle. At baseline levels on TCP, during the active phase, patient cells moved at significantly smaller turn angles than control cells (Patient: 78.29 ± 0.76 degrees, *n* = 5; Control: 81.86 ± 1.07 degrees, *n* = 5) (Figure 2C; *p* = 0.027, *t* = 2.71, df = 8). During the idling phase, there was no significant difference in the turn angles of patient cells and control cells (Figure 2D; *p* = 0.22, *t* = 1.33, df = 8).

### 2.6. Patient Cell Trajectories Were Straighter on Most Concentrations of Extracellular Matrix Proteins

**Figure 3:**
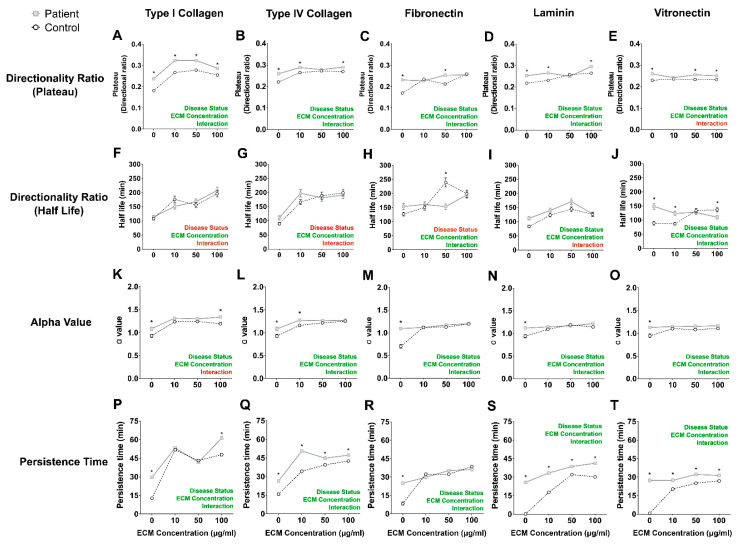
Patient cell migration is more persistent than control cell migration on most ECM protein substrates. (**A**–**E**): Directionality Ratio Plateau values for patient and control cells migrating on the ECM proteins at different concentrations. (**F**–**J**): Directionality Ratio Half-life values for patient and control cells migrating on the ECM proteins at different concentrations. (**K**–**O**): MSD α-values for patient and control cells migrating on the ECM proteins at different concentrations. (**P**–**T**): Persistence times for patient and control cells migrating on the ECM proteins at different concentrations. All data are presented as the mean track distance ± SEM. Control cells: open circles with black dotted lines (*n* = 9 independent cell lines). Patient cells: closed grey squares with grey solid lines (*n* = 9 independent cell lines). Two-way ANOVA was used to compute the main effects of disease status, ECM protein concentration and whether an interaction exists between the two main effects. Summary of statistical test is presented in each graph as “Disease status”, “ECM concentration” and “Interaction”; red: *p* > 0.05, green *p* < 0.05. Detailed ANOVA results are presented in Tables S1–S4. * = *p* < 0.05, Tukey post hoc tests. In general, the concentration–response curves for the patient cells tended to be flatter than for the control cells on most substrates, indicating a lesser sensitivity of patient cells to the protein substrates.

Cells migrated on different ECM proteins at four concentrations (0, 10, 50, and 100 µg/mL). Time-dependent changes to the directionality ratio were calculated for each cell (Figure S1) by fitting the data to a one-phase decay function to quantify two parameters: Directionality Ratio Plateau (DRPlateau) and Directionality Ratio Half-life (DRHalf-life, Figure 3). For each parameter and ECM protein, two-way ANOVA was performed to compare the main effects of disease status and ECM concentration and their interaction (Tables S1 and S2). For DRPlateau, there was a significant effect of disease status for all ECM proteins and a significant effect of ECM concentration for all ECM proteins except vitronectin (Table S1). Multiple comparison Tukey tests were performed post hoc to compare patient and control values. There were significant disease status–ECM concentration interactions for all ECM proteins (Table S1). For all ECM proteins, patient cells had a higher Plateau than control cells (* in Figure 3A–J), with the largest differences between patient and control cells on collagen I. For DRHalf-life, there was a significant effect of disease status only for laminin and vitronectin (Figure 3F–J). Half-life increased with ECM concentration for patient and control cells on all ECM proteins (Table S2). There were significant disease status–ECM concentration interactions only for fibronectin and vitronectin (Table S2). Multiple post hoc comparison Tukey tests indicated a significant difference between patient and control cells for 50 µg fibronectin and all except 50 µg for vitronectin (Figure 3I,J). 

### 2.7. Patient Cells Explored Larger Surface Areas on Most Concentrations of ECM Proteins

The area explored by each cell was quantified by calculating mean square displacement (MSD) plotted against time and presented as means for each patient and control at each ECM concentration (Figure S1F–J). These MSD–time curves were transformed into log–log curves, and the slope of the log–log curve, α, was calculated for each ECM protein (Figure 3K–O). Two-way ANOVA of the α values compared the main effects of disease status and ECM protein concentration and their interaction. On all ECM proteins, both disease status and ECM protein concentration were significant (*p* < 0.05, Table S3), with significant interactions between the two main effects for all ECM proteins except collagen I (Table S3). Multiple comparison Tukey post hoc tests indicated significant differences between patient and control cells on different ECMs, with all ECMs different 0 µg/mL (* in Figure 3), although the differences with concentration were slight. 

### 2.8. Patient Cells Persisted for Longer on Most Concentrations of Extracellular Matrix Proteins

Persistence time was estimated from the inverse of the decay rate of the one-phase decay function fitted to the velocity autocorrelation (Figure S1K–O, Figure 3P–T). A two-way ANOVA test indicated significant main effects of disease status and ECM concentrations and their interactions for all ECM proteins (Table S4). Post hoc Tukey tests indicated significant disease status differences at most ECM concentrations (* in Figure 3P–T). As a measure of directional persistence, persistence time showed the most consistent patient–control differences.

### 2.9. Patient Cell Active and Idling Times Were Similar on Most Extracellular Matrix Proteins

**Figure 4:**
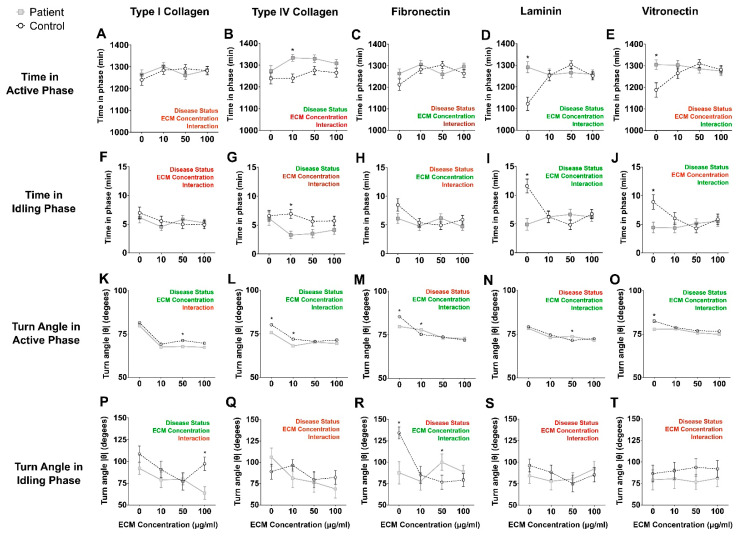
Patient cell and control cell active and idling phases and turn angles were similar on different ECM protein substrates. (**A**–**E**): Time in Active Phase values for patient and control cells migrating on the ECM proteins at different concentrations. (**F**–**J**): Time in Idling Phase values for patient and control cells migrating on the ECM proteins at different concentrations. (**K**–**O**): Turn Angles after Active Phase for patient and control cells migrating on the ECM proteins at different concentrations. (**P**–**T**): Turn Angles after Idling Phase for patient and control cells migrating on the ECM proteins at different concentrations. All data are presented as the mean track distance ± S.E.M. Control cells: open circles with black dotted lines (*n* = 9 independent cell lines). Patient cells: closed grey squares with grey solid lines (*n* = 9 independent cell lines). Two-way ANOVA was used to compute the main effects of: disease status, ECM protein concentration and whether an interaction exists between the two main effects. Summary of statistical test is presented in each graph as “Disease status”, “ECM concentration” and “Interaction”; red: *p* > 0.05, green *p* < 0.05. Detailed ANOVA results are presented in Supplementary Tables S4–S8. * = *p* < 0.05, Tukey post hoc tests.

The durations of the active and idling phases were generally similar for patient and control cells with differences at only a few ECM concentrations (*, Figure 4A–J). Two-way ANOVA of Active Phase Time indicated significant effects of disease status for collagen IV and laminin (Table S4) and significant effects of ECM concentration for fibronectin and laminin (Table S5). Two-way ANOVA of Idle Phase Time indicated significant effects of disease status for collagen IV, laminin, and vitronectin (Table S5) and significant effects of ECM concentration for fibronectin and laminin (Table S5). 

### 2.10. Patient Cell Turn Angles Were Similar on Most Extracellular Matrix Proteins

The turn angles in active and idling phases were generally similar for patient and control cells, with differences only at a few ECM concentrations (*, Figure 4K–T). The turn angles of patient and control cells reduced with increasing ECM concentrations (Figure 4K–T). Two-way ANOVA indicated significant effects of disease status for collagen I in both active and idle phases and collagen IV and vitronectin in the active phase (Tables S7 and S8). ECM concentration was significant for all ECMs in the active phase and for collagen I and fibronectin in the idle phase. Interactions were significant for all ECMs except collagen I in the active phase and only fibronectin in the idle phase (Tables S7 and S8).

## 3. Discussion

**Figure 5:**
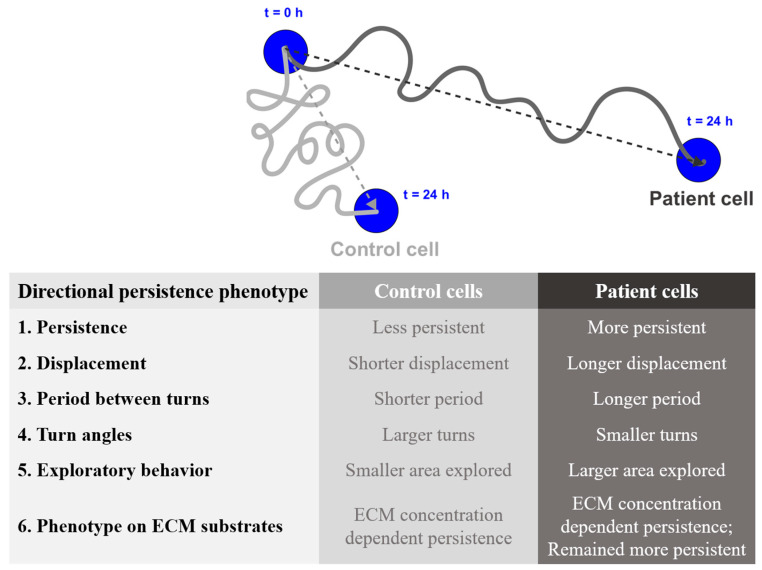
Summary of findings.

In this study, we demonstrate that schizophrenia patient-derived cells were more persistent in their migration than controls, that is, they traveled for longer in a straight line before turning and then made smaller angled turns. This difference was seen at baseline, when migrating on uncoated tissue culture plastic, and on multiple ECM proteins at different concentrations. On plastic they had straighter trajectories, explored larger areas, spent more time in persistent movement, spent more time in active movement, and made smaller turns compared to control cells (Figure 5). When migrating on different ECM protein substrates, the intrinsic bias of patient cell persistence was maintained. This is in contrast to their speed, which was independent of substrate concentrations [18], further supporting the hypothesis that the cellular mechanisms regulating the speed and persistence of cell migration are independent in these cells [20]. The intrinsic bias in directional persistence in patient cells and their lack of migration speed regulation on ECM proteins could plausibly alter the characteristics of neuronal migration trajectories during brain development in schizophrenia. 

The cellular mechanisms responsible for this migration bias are likely complex, but must involve processes interposed between the detection of the ECM and the generation of cell movement. Cells interact with the extracellular microenvironment through the selective binding of integrin receptors to specific substrate proteins, which ultimately controls whether a cell adheres and moves in that microenvironment [23]. The patient cells of this study have the integrins to detect the ECMs used here [18]. Transcriptome analysis of the same patient and control cells growing on tissue culture plastic revealed altered expression of multiple genes in patient cells in relevant cell signaling pathways, in particular, significant dysregulation of the expression of key regulatory genes that make up the FAK signaling complex, including *PIK3CD*, *PIK3R1*, *PRKCA, SRC, ROCK2, CRK, FYN*, and *RAC3* [19], although its protein expression is the same in patient and control cells [24]. Patient cells also have altered expression of *FRMD3*, *FRMD6,* and *PLEKHC1/FERMT2* genes from the FERM domain, a “switch” in the FAK complex, which regulates biophysical forces through tension forces exerted by F-actin [25]. The patient cells have reduced expression of key genes that make up the Arp2/3 complex, *ARPC2, ARPC4,* and *ARPC5L* [19]. Protein expressions of members of the ARP2/3 complex are all downregulated in patient cells (ARPC1A, ARPC1B, ARPC2, ARPC3, ARPC4, ARPC5, and ARPC5L), although only ARPC2 downregulation reached statistical significance [24]. During cell migration, Arp2/3 regulates actin branching in the lamellipodia to control the directionality and persistence [26,27], as well as turning and pausing tendencies [22]. ARP2/3 transcript levels are reduced in the prefrontal cortex in the post-mortem brain in schizophrenia, as are nucleation promotion factors that regulated ARP2/3 (cortactin, N-WASP) and are suggested to contribute to dendritic spine loss in that region [28]. Other pathways affected in patient cells were “Signaling of Rho Family GTPases” (transcription of 38 genes affected), “Regulation of Actin-based Motility by Rho” (16 genes), and “Actin Nucleation by ARP-WASP Complex” (13 genes). Rho signaling stabilizes microtubules [29] and regulates F-actin flow to relay traction force signals to the focal adhesions [30]. “Actin Cytoskeleton Signaling” (30 genes) was also dysregulated. These signaling pathways work in synchrony to orchestrate cell polarization and the sense of directionality in an actively moving cell [31]. Integrin receptor binding initiates the formation of focal adhesions, which relay force alterations from the pulling and tugging of cytoskeletal microtubules and F-actin to membrane-bound integrin receptors [27,32]. These forces dictate mechanistic changes in movement directionality [33], directional persistence [34], and coordinated turning of cells [35]. These gene expression differences in patient and control cells will be useful to guide hypotheses for the molecular regulation of the biases in directional persistence and cell speed in patient cells.

Patient-derived cell-based models provide useful systems for investigating dynamic cell behaviors in cell migration in schizophrenia that cannot be achieved in animal models or post-mortem studies. Other schizophrenia-associated cell migration phenotypes have been reported as reduced invasion of patient olfactory neural precursors, measured by transwell invasion assay [36]; reduced neurosphere size in outgrowth assays for patient hiPSC neural progenitor cells [12,37]; and increased closure of scratch wound assay by patient hiPSC neural progenitor cells [12]. Patient-derived ONS cells have multiple other phenotypes that relate to cell migration: less adhesive, smaller and fewer focal adhesions, faster focal adhesion dynamics, cell migration speed independent of ECM protein concentration [17,18], and disrupted reelin signaling [38]. Through dynamic investigations of cell migration behavior, the current study has revealed biased directional persistence in patient cells compared to control cells at baseline and on ECM proteins at different concentrations. The sources of cell migration biases in directional persistence and speed likely arise in the complex regulatory pathways interposed between the detection of the ECM proteins via integrin receptors, the formation of focal adhesions, lamellipodia formation, actin branching and elongation, and the actin flow generating movement. Patient-derived ONS cells provide the tools to investigate these dynamic functions and the key regulatory pathways (genes, proteins, phosphorylation status) to dissect the molecular events involved in the regulatory biases of cell migration in schizophrenia and potentially to identify the genes and proteins involved in this dysregulation.

Well-orchestrated neuronal migration is vitally important during brain development to guide different neural cell types to correct parts of the brain at the correct time points. During early embryogenesis, stem cells are directed through signals and cues to undertake directionally persistent migration to various regions of the embryo, which triggers speciation into different organs [1,2,3]. In the context of brain development, neural cells move persistently for long distances as directed by their surrounding ECM substrate [5,39] and soluble factors [40,41,42]. Neural cells also frequently undergo directional changes through well-coordinated turning events to re-orientate their positioning [6,7,43]. Structural changes are evident in post-mortem brains from schizophrenia patients because of cell migration deficits; for example, aberrant interneuron migration [10]. Different gene expression analyses of post-mortem brain samples and stem cells derived from schizophrenia patients repeatedly implicate dysregulation gene pathways relevant to cell migration [11,12,13,14], which is supported by defective cell migration observed in vitro in patient-derived stem cells and brain organoids [12,15]. Our current work provides new insight into the dynamic migration deficits of cells from schizophrenia patients observed as dysfunctional directional persistence, which offers a mechanistic explanation to the cell migration phenotype observed previously by us and others. Defects to directional persistence in our patient ONS cell-based model give us clues into how cell-intrinsic differences during cell migration could cause incorrect positioning and wiring of the human brain during neurodevelopment, finally leading to the onset of schizophrenia.

To conclude, new findings presented in this work have deepened our understanding of previously observed cell migration deficits in schizophrenia patient-derived cells. We uncovered disease-dependent differences in the directional persistence of patient cells, in parallel to migration speed unresponsiveness to the ECM microenvironment [18]. The aberrant cell migration phenotype established by others and us opens the door to further investigations into specific risk genes and signaling pathways that may play a role as drivers for cell migration, which will ultimately help us understand the biology of schizophrenia.

## 4. Materials and Methods

### 4.1. Olfactory Neurosphere-Derived Cells

Patient-derived and control-derived ONS cells used in this study are the same as those used previously for cell migration studies [17,18,38]. They were generated from olfactory mucosal biopsies derived from patients with schizophrenia (*n* = 9) and age-matched healthy participants (*n* = 9) [19]. ONS cells derived from participants with schizophrenia were referred to as “patient cells”, while cells from healthy participants were referred to as “control cells”. The patient cohort was selected based on disease classification using the Diagnostic Interview for Psychosis (DIP), according to the Diagnostic and Statistical Manual for Mental Disorders IV (DSM-IV). All biopsies were approved by the Ethics Committee for West Moreton Region, Queensland Health and the Griffith University Human Ethics Committee (Brisbane, Queensland, Australia). All participants gave written, informed consent for their cells to be grown in vitro, banked, and used for experiments to understand the biological bases of schizophrenia. All experiments were conducted according to guidelines from the National Health and Medical Research Council of Australia. Patient details are published [18]. Details of cell culture are published [18,19]. 

### 4.2. High-Throughput Single Cell Tracking

Datasets in this study were derived from the same high-throughput image sequence acquisition used to compute cell migration speeds [18]. Details of the cell tracking assay are published [18]. Briefly, cell cycle-synchronized ONS cells were seeded into wells of 96-well plates (CellCarrier plates, Perkin Elmer, Waltham, MA, USA), which were coated with different concentrations of extracellular matrix proteins (ECMs): collagen I, collagen IV, fibronectin, laminin, and vitronectin [18]. Cells were also seeded on uncoated tissue culture plastic (TCP) that represents culture conditions at baseline prior to coating of abovementioned ECM substrate proteins. Cells (2500 cells per well) were incubated with a nucleus stain (NucBlue Live ReadyProbes reagent, Molecular Probes, Life Technologies, Grand Island, NY, USA). Live single cells were imaged every 30 min for 24 h (10× objective, 360–400 nm excitation wavelength). Ten or 11 fields of view were imaged for each ECM concentration for each patient and control ONS cell line. Images were acquired and analyzed on the Operetta High Content Imaging System (Perkin Elmer, Llantrisant, UK) using Harmony High Content Imaging and Analysis Software (Perkin Elmer). Raw datasets were imported into Microsoft Excel (Microsoft, Redmond, WA, USA) and were pre-organized into data columns that contain information about cell identification (I.D.), well I.D., fields of view I.D., and X- and Y-displacements (in µm) across all acquired time points. Only cells which remained in the field of view for the full 24 h period were included in the analysis. Cells with incomplete data were removed from the final datasets to reduce bias and to maintain consistency and reproducibility, resulting in an average of 202 ± 17 cells assessed for each ECM concentration and group (8069 cells in total).

### 4.3. In Silico Single Cell Directional Persistence Analysis

The open source program DiPer developed by Gorelik and Gautreau [20] was used to quantify different measures of directional persistence in control and patient cells using the dataset acquired from the high-throughput tracking assay. Cell track datasets were organized according to the developer’s protocol, where Excel columns would start with cell identifiers, followed by a column with sequential numerical increments of 47 time frames (i.e., *n* = 0, 1, 2, 3, …, 47), and the X and Y displacement coordinates across all frames for a total duration of 24 h. Measured DiPer parameters included directionality ratio (DRPlateau, DRHalf-life), mean squared displacement (MSD), and persistence time (inverse of rate of velocity autocorrelation), which were published as Visual Basic for Applications source codes [20] under the file names “DirRatio.txt”, “MSD.txt”, and “Vel_Cor.txt”, respectively. All DiPer program source codes were inserted into Microsoft Excel as Macro “Modules” via “Visual Basic” function in the “Developer” module. Two different parameters were computed to quantify directional persistence: α-values and persistence times. Datasets produced from each DiPer program were imported into GraphPad Prism (version 6.05, GraphPad Software, Inc., La Jolla, CA, USA) to build graphs and plots for this study. 

The following mathematical formulas were used to calculate each DiPer parameter [20]:

$$Directionality\;ratio=\frac{Cell\;displacement}{Cell\;accumulated\;distance}$$

$$Mean\;squared\;displacement,\;MSD\left(n\right)=\frac{1}{N-n+1}\sum_{i=0}^{N-n}[{\left(x_{\left(i+n\right)∆t}-x_{i∆t}\right)}^{2}+{\left(y_{\left(i+n\right)∆t}-y_{i∆t}\right)}^{2}]$$

$$Velocity\;autocorrelation,v_{ac}\left(n\right)=\;\frac{1}{N-n}\left(\sum_{i=0}^{N-n}v_{i}\times v_{i+n}\right)\times \frac{1}{N}\sum_{i=0}^{N-1}{\left|v_{i}\right|}^{2}$$

*N*: total tracks measured, *n*: frame number, *x*: X displacement coordinate, *y*: Y displacement coordinate, ∆*t*: time interval, *v_i_*: autocorrelated velocity vector between two subsequent time frames.

### 4.4. In Silico Single Cell Pausing and Turning Analysis

The tendency to pause or turn during cell migration was computed with an open source program [22]. Cell track datasets were organized according to the developer’s protocol, where Excel columns would start with cell identifiers, followed by a column with sequential numerical increments of 47 time frames (i.e., t = 0, 1, 2, 3, …, 47), and the X and Y displacement coordinates across all time frames over the total tracked duration of 24 h. 

Cell movements were categorized into two phases—active migration phase and idling/pausing phases. A speed threshold constant (*v_thr_*) was first computed to identify frames where the cell paused (idling phase) and when cells were moving (active phase), using the formula $v_{thr}=\left(2-\alpha \right)\times v$, where *v* is the average cell speed for the whole trajectory (24 h) and α is the value computed from the slope of the log–log MSD curve. Cells with instantaneous displacement values smaller than the *v_thr_* were considered to be in the idling phase. Cells that displaced at equal or greater value than the *v_thr_* were moving in the active phase. “Phase_Duration” source code was used to calculate the time each tracked cell spends in the active and idling phases, expressed as a measure of time (min). The cell turning program contains a sequence of source codes, to compute turn angles at all active and idling phases during cell movement, where the program sequence is as follows using published source codes: “Theta_101” source code used to identify phase types and compute angle change for all phases, “Theta_Abs_Value” used to take absolute values regardless of if it is a negative or positive readout, and “Theta_Concatenate” completed as a quality control step to prepare datasets ready for statistical analyses. Turn angles were reported as absolute angles (θ).

### 4.5. Statistical Analysis

All statistical analyses presented in this paper were performed using GraphPad Prism (version 6.05, GraphPad Software, Inc., La Jolla, CA, USA). All data were presented as mean averages of *n* = 9 patient cell lines and *n* = 9 healthy control cell lines. All error bars represent standard error of the mean (S.E.M.). Parametric, two-tailed Student’s *t*-test was used to estimate significance of pair-wise comparisons of control cell and patient cell datasets. Two-way analysis of variance (ANOVA) tests were conducted on datasets with two variables (disease status and ECM protein concentration) to estimate main effects and interaction of variables on dependent variables. Multiple comparisons Tukey test was performed post hoc to find deviations between two groups (disease status and/or ECM protein concentration). Curve fitting functions were used to estimate changes to time-dependent and concentration-dependent variables. Directionality ratio and velocity autocorrelation graphs were fitted according to a one-phase exponential decay function to estimate the plateau and half-life, and persistence time (1/decay rate), respectively. One-phase decay function: $Y=\left(Y_{0}-Plateau\right)\times \exp\left(-K\times X\right)+Plateau,$ where *Y*_0_ is *Y*-axis intercept and *K* is the rate of decay. Linear regression function was used to estimate the slope/variable rate of change for log–log MSD graphs and all concentration-dependent changes to measured variables. Datasets were fitted using the least squares (ordinary) fit to the linear regression function: $Y=KX+Y_{0},$ where *K* is the slope/rate of change and *Y*_0_ is the *Y*-axis intercept. Extra sum-of-squares F test was conducted to estimate if the resulting slope was positive, negative, or zero value (no slope), and to estimate differences between the control and patient slopes. All statistical tests were considered significant if the *p*-value was less than or equal to α = 0.05.

## Data Availability

Data not contained in the paper or Supplementary Materials are available from the corresponding author.

## Supplementary Materials

The following are available online at https://www.mdpi.com/article/10.3390/ijms22179177/s1.
