# Supplementary material for: Directional Persistence of Cell Migration in Schizophrenia Patient-Derived Olfactory Cells
Source: Int J Mol Sci. 2021 Aug 25;22(17):9177. doi: 10.3390/ijms22179177 (PMC8430705; doi:10.3390/ijms22179177)
Supplement: Supplementary file 1 [file ijms-22-09177-s001.zip › ijms-1343218-supplementary.pdf]

International Journal of *Molecular Sciences*

MDPI

Supplementary Materials

Supplementary Figure S1. Persistence measures of Patient and Control cells on different ECMs. Data at each time point are mean and SEM. Patient cells in red. Control cells in blue. Derived parameters were calculated from these plots: Directionality Ratio (DRPlateau, DRHalf-life); Mean Square Displacement (MSD α); Velocity autocorrelation (Persistence time).

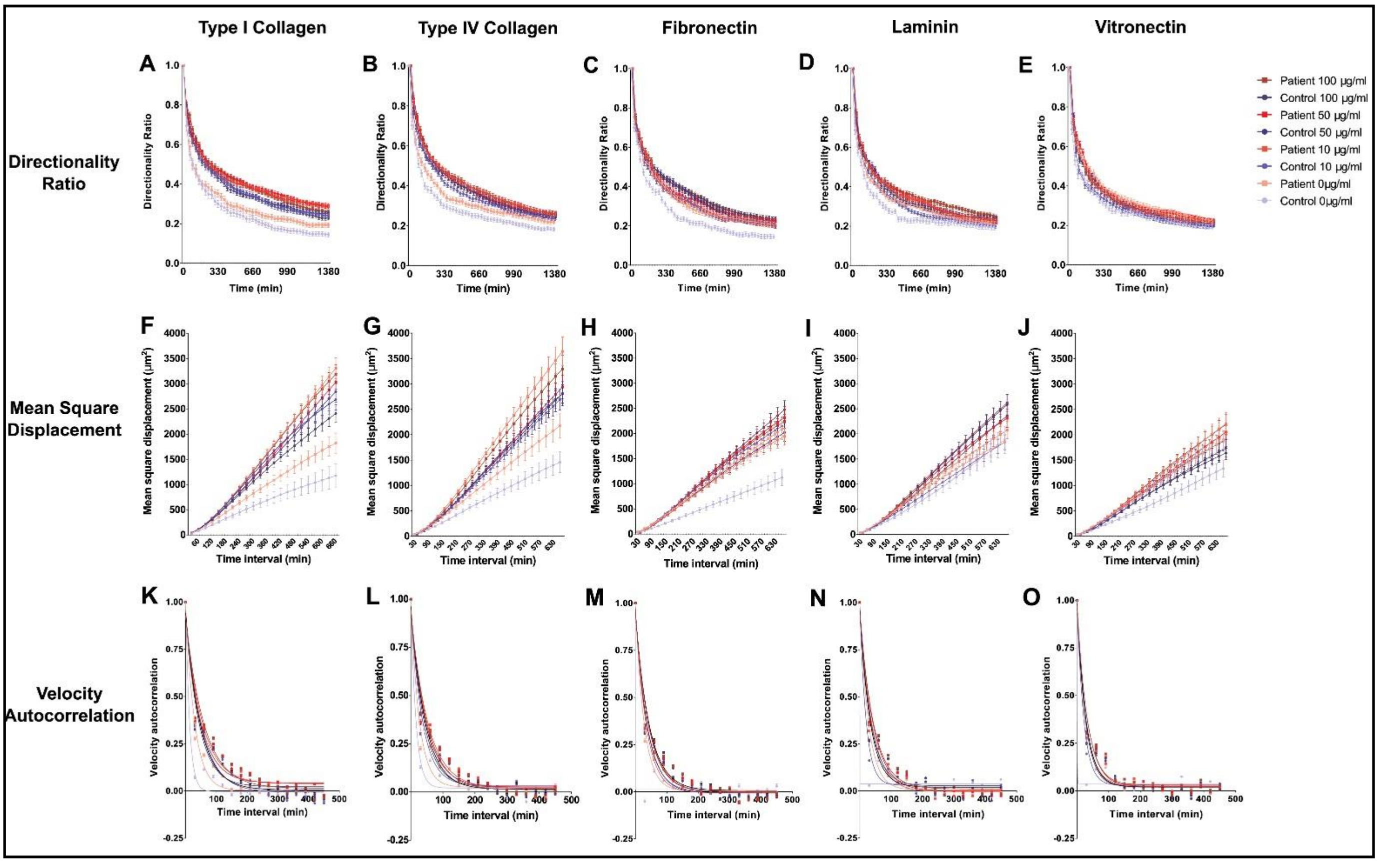

Supplementary Tables

Supplementary Table S1. Summary of two-way ANOVAs estimating effects of Disease Status and ECM protein concentration on DRPlateau. Significant factorial effects are highlighted in green (*P*<0.05) and non-significant factorial effects are highlighted in grey (*P*>0.05).

ECM Protein	Disease status			ECM concentration			Interaction		
	F	***P***	**R^2**	***F***	***P***	**R^2**	***F***	***P***	**R^2**
**Type I Collagen**	$F(1, 1841) = 427.4$	< 0.001	13.11	$F(3, 1841) = 324.1$	< 0.0001	29.83	$F(3, 1841) = 6.26$	0.0003	0.58
**Type IV Collagen**	$F(1, 1622) = 74.20$	< 0.0001	3.96	$F(3, 1622) = 52.52$	< 0.0001	8.41	$F(3, 1622) = 6.48$	0.0002	1.04
**Fibronectin**	$F(1, 1447) = 69.50$	< 0.0001	3.77	$F(3, 1447) = 71.17$	< 0.0001	11.57	$F(3, 1447) = 38.56$	< 0.0001	6.27
**Laminin**	$F(1, 1789) = 75.78$	< 0.0001	3.67	$F(3, 1789) = 51.14$	< 0.0001	7.43	$F(3, 1789) = 15.78$	< 0.0001	2.29
**Vitronectin**	$F(1, 1330) = 88.63$	< 0.0001	6.15	$F(3, 1330) = 1.75$	0.155	0.36	$F(3, 1330) = 5.85$	0.0006	1.22

Supplementary Table S2. Summary of two-way ANOVAs estimating effects of Disease Status and ECM protein concentration on DRHalf-life. Significant factorial effects are highlighted in green (*P*<0.05) and non-significant factorial effects are highlighted in grey (*P*>0.05).

ECM Protein	Disease status			ECM concentration			Interaction		
	F	***P***	**R^2**	***F***	***P***	**R^2**	***F***	***P***	**R^2**
**Type I Collagen**	$F(1, 1841) = 0.02$	0.889	0.001	$F(3, 1841) = 25.83$	< 0.0001	4.03	$F(3, 1841) = 1.45$	0.225	0.23
**Type IV Collagen**	$F(1, 1622) = 1.40$	0.238	0.08	$F(3, 1622) = 29.73$	< 0.0001	5.19	$F(3, 1622) = 1.55$	0.201	0.27
**Fibronectin**	$F(1, 1447) = 2.16$	0.142	0.14	$F(3, 1447) = 10.72$	< 0.0001	2.14	$F(3, 1447) = 8.07$	< 0.0001	1.61
**Laminin**	$F(1, 1789) = 11.50$	0.0007	0.62	$F(3, 1789) = 20.90$	< 0.0001	3.36	$F(3, 1789) = 1.30$	0.274	0.21
**Vitronectin**	$F(1, 1330) = 9.18$	0.003	0.66	$F(3, 1330) = 4.02$	0.007	0.87	$F(3, 1330) = 14.01$	< 0.0001	3.02

Supplementary Table S3. Summary of two-way ANOVAs estimating effects of Disease Status and ECM protein concentration on MSD α. Significant factorial effects are highlighted in green (*P*<0.05) and non-significant factorial effects are highlighted in grey (*P*>0.05).

ECM Protein	Disease status			ECM concentration			Interaction		
	F	***P***	**R^2**	***F***	***P***	**R^2**	***F***	***P***	**R^2**
**Type I Collagen**	$F(1, 1587) = 44.77$	< 0.0001	2.44	$F(3, 1587) = 66.15$	< 0.0001	10.80	$F(3, 1587) = 2.35$	0.071	0.38
**Type IV Collagen**	$F(1, 1474) = 22.57$	< 0.0001	1.38	$F(3, 1474) = 44.35$	< 0.0001	8.12	$F(3, 1474) = 3.07$	0.027	0.56
**Fibronectin**	$F(1, 1447) = 36.54$	< 0.0001	2.12	$F(3, 1447) = 53.33$	< 0.0001	9.28	$F(3, 1447) = 27.15$	< 0.0001	4.72
**Laminin**	$F(1, 1789) = 18.95$	< 0.0001	1.01	$F(3, 1789) = 19.11$	< 0.0001	3.04	$F(3, 1789) = 6.49$	0.0002	1.03
**Vitronectin**	$F(1, 1330) = 25.45$	< 0.0001	1.84	$F(3, 1330) = 6.13$	0.0004	1.33	$F(3, 1330) = 3.17$	0.024	0.69

Supplementary Table S4. Summary of two-way ANOVAs estimating effects of Disease Status and ECM protein concentration on Persistence Time. Significant factorial effects are highlighted in green (P<0.05) and non-significant factorial effects are highlighted in grey (P>0.05).

ECM Protein	Disease status			ECM concentration			Interaction		
	F	***P***	**R^2**	***F***	***P***	**R^2**	***F***	***P***	**R^2**
**Type I Collagen**	$F(1, 1841) = 107.5$	< 0.0001	3.18	$F(3, 1841) = 441.0$	< 0.0001	39.11	$F(3, 1841) = 37.09$	< 0.0001	3.29
**Type IV Collagen**	$F(1, 1622) = 207.60$	< 0.0001	7.47	$F(3, 1622) = 298.90$	< 0.0001	32.26	$F(3, 1622) = 17.76$	< 0.0001	1.92
**Fibronectin**	$F(1, 1447) = 34.65$	< 0.0001	1.54	$F(3, 1447) = 206.6$	< 0.0001	27.49	$F(3, 1447) = 51.32$	< 0.0001	6.83
**Laminin**	$F(1, 1789) = 874.9$	< 0.0001	20.54	$F(3, 1789) = 463.5$	< 0.0001	32.65	$F(3, 1789) = 68.13$	< 0.0001	4.80
**Vitronectin**	$F(1, 1330) = 463.00$	< 0.0001	17.86	$F(3, 1330) = 178.60$	< 0.0001	20.47	$F(3, 1330) = 96.51$	< 0.0001	11.06

Supplementary Table S5. Summary of two-way ANOVAs estimating effects of Disease Status and ECM protein concentration on Duration in Active Phase. Significant factorial effects are highlighted in green (P<0.05) and non-significant factorial effects are highlighted in grey (P>0.05).

ECM Protein	Disease status			ECM concentration			Interaction		
	F	***P***	**R^2**	***F***	***P***	**R^2**	***F***	***P***	**R^2**
**Type I Collagen**	$F(3, 1841) = 0.06$	0.807	0.03	$F(3, 1841) = 1.63$	0.181	0.26	$F(3, 1841) = 0.79$	0.50	0.13
**Type IV Collagen**	$F(3, 1622) = 15.22$	< 0.0001	0.92	$F(3, 1622) = 1.87$	0.1332	0.34	$F(3, 1622) = 0.83$	0.476	0.15
**Fibronectin**	$F(3, 1447) = 1.28$	0.257	0.08	$F(3, 1447) = 2.98$	0.031	0.61	$F(3, 1447) = 2.14$	0.093	0.44
**Laminin**	$F(3, 1789) = 4.76$	0.029	0.26	$F(3, 1789) = 3.87$	0.009	0.64	$F(3, 1789) = 7.71$	< 0.0001	1.27
**Vitronectin**	$F(3, 1330) = 3.73$	0.054	0.28	$F(3, 1330) = 1.88$	0.132	0.42	$F(3, 1330) = 3.86$	0.009	0.86

Supplementary Table S6. Summary of two-way ANOVAs estimating effects of Disease Status and ECM protein concentration on Duration in Idle Phase. Significant factorial effects are highlighted in green (P<0.05) and non-significant factorial effects are highlighted in grey (P>0.05).

ECM Protein	Disease status			ECM concentration			Interaction		
	F	***P***	**R^2**	***F***	***P***	**R^2**	***F***	***P***	**R^2**
**Type I Collagen**	$F(3, 1841) = 0.09$	0.767	0.004	$F(3, 1841) = 1.60$	0.187	0.26	$F(3, 1841) = 0.64$	0.589	0.10
**Type IV Collagen**	$F(3, 1622) = 11.18$	0.0008	0.68	$F(3, 1622) = 1.75$	0.154	0.32	$F(3, 1622) = 1.28$	0.280	0.23
**Fibronectin**	$F(3, 1447) = 1.57$	0.210	0.11	$F(3, 1447) = 2.99$	0.030	0.61	$F(3, 1447) = 1.65$	0.177	0.34
**Laminin**	$F(3, 1789) = 4.10$	0.043	0.22	$F(3, 1789) = 2.72$	0.043	0.45	$F(3, 1789) = 7.72$	< 0.0001	1.27
**Vitronectin**	$F(3, 1330) = 4.55$	0.033	0.34	$F(3, 1330) = 1.61$	0.185	0.36	$F(3, 1330) = 3.00$	0.030	0.67

Supplementary Table S7. Summary of two-way ANOVAs estimating effects of Disease Status and ECM protein concentration on Turn Angle in Active Phase. Significant factorial effects are highlighted in green (*P*<0.05) and non-significant factorial effects are highlighted in grey (*P*>0.05).

ECM Protein	Disease status			ECM concentration			Interaction		
	F	***P***	**R^2**	***F***	***P***	**R^2**	***F***	***P***	**R^2**
**Type I Collagen**	F(1, 86845) = 38.95	<0.0001	0.04	F(3, 86845) = 261.9	<0.0001	0.90	F(3, 86845) = 1.28	0.28	0.004
**Type IV Collagen**	F(1, 72363) = 46.12	< 0.0001	0.06	F(3, 72363) = 89.88	<0.0001	0.37	F(3, 72363) = 5.17	0.001	0.02
**Fibronectin**	F(1, 64408) = 2.17	0.141	0.003	F(3, 64408) = 107.8	<0.0001	0.50	F(3, 64408) = 16.77	<0.0001	0.08
**Laminin**	F(1, 79731) = 0.74	0.400	0.0009	F(3, 79731) = 63.74	<0.0001	0.24	F(3, 79731) = 4.92	0.002	0.02
**Vitronectin**	F(1, 59389) = 21.91	<0.0001	0.04	F(3, 59389) = 18.94	<0.0001	0.09	F(3, 59389) = 3.71	0.011	0.019

Supplementary Table S8. Summary of two-way ANOVAs estimating effects of Disease Status and ECM protein concentration on Turn Angle in Idlng Phase. Significant factorial effects are highlighted in green (*P*<0.05) and non-significant factorial effects are highlighted in grey (*P*>0.05).

ECM Protein	Disease status			ECM concentration			Interaction		
	F	***P***	**R^2**	***F***	***P***	**R^2**	***F***	***P***	**R^2**
**Type I Collagen**	F(1, 393) = 6.55	0.011	1.59	F(3, 393) = 2.94	0.033	2.13	F(3, 393) = 1.67	0.173	1.21
**Type IV Collagen**	F(1, 314) = 0.26	0.607	0.08	F(3, 314) = 2.05	0.106	1.90	F(3, 314) = 1.08	0.358	1.00
**Fibronectin**	F(1, 341) = 0.63	0.429	0.17	F(3, 341) = 4.15	0.007	3.37	F(3, 341) = 5.21	0.002	4.23
**Laminin**	F(1, 351) = 0.17	0.681	0.05	F(3, 351) = 0.85	0.470	0.72	F(3, 351) = 0.66	0.575	0.56
**Vitronectin**	F(1, 257) = 2.29	0.131	0.88	F(3, 257) = 0.04	0.988	0.05	F(3, 257) = 0.08	0.969	0.10
